# Mapping land cover change over continental Africa using Landsat and Google Earth Engine cloud computing

**DOI:** 10.1371/journal.pone.0184926

**Published:** 2017-09-27

**Authors:** Alemayehu Midekisa, Felix Holl, David J. Savory, Ricardo Andrade-Pacheco, Peter W. Gething, Adam Bennett, Hugh J. W. Sturrock

**Affiliations:** 1 Malaria Elimination Initiative, Global Health Group, University of California San Francisco, San Francisco, California, United States of America; 2 Big Data Institute, Nuffield Department of Medicine, University of Oxford, Oxford, United Kingdom; Bristol University/Remote Sensing Solutions Inc., UNITED STATES

## Abstract

Quantifying and monitoring the spatial and temporal dynamics of the global land cover is critical for better understanding many of the Earth’s land surface processes. However, the lack of regularly updated, continental-scale, and high spatial resolution (30 m) land cover data limit our ability to better understand the spatial extent and the temporal dynamics of land surface changes. Despite the free availability of high spatial resolution Landsat satellite data, continental-scale land cover mapping using high resolution Landsat satellite data was not feasible until now due to the need for high-performance computing to store, process, and analyze this large volume of high resolution satellite data. In this study, we present an approach to quantify continental land cover and impervious surface changes over a long period of time (15 years) using high resolution Landsat satellite observations and Google Earth Engine cloud computing platform. The approach applied here to overcome the computational challenges of handling big earth observation data by using cloud computing can help scientists and practitioners who lack high-performance computational resources.

## Introduction

Quantifying and monitoring the spatial and temporal dynamics of the global land use land cover (LULC) is essential for better understanding many of the Earth’s land surface processes. Human-induced land cover changes may affect land surface processes including urbanization, drought, and flood which impact the world’s population [1, 2]. Understanding these changes can allow quantifying and monitoring trends in agriculture [3], fresh water resources [4], forest cover [5–7], and disease transmission [8, 9]. Lack of high spatial resolution (30 m) and regularly updated LULC data limit our ability to quantify the spatial extent and monitor the temporal dynamics of these changes. Earlier attempts to generate continental-scale LULC products were limited to coarse spatial scale (250m-1km) which lacked sufficient spatial details [10–13].

There is a need for a high spatial resolution (30 m) and regularly updated LULC data to improve our understanding of changes in the land surface processes. However, there are challenges in generating global to continental scale LULC maps from high spatial resolution Landsat observations that span over a long period of time. These challenges have included the lack of freely available earth observation data and high-performance computing to process and analyze these data. Starting from 2008, the United States Geological Survey (USGS) has been distributing Landsat data at no cost for public use. This created an opportunity for scientists and practitioners to use Landsat satellite data to map and monitor LULC at continental to global scale and over a long time series [5, 7, 14]. Since the USGS released the Landsat data archive for public use, there has been an increasing trend in efforts to map land use land cover using these data [15–18]. However, most of these efforts have been limited to local scale analyses due to the computational requirements of analyzing large volumes of data. For example, nearly ~10,000 Landsat scenes or ~3 terabytes are required to produce a global land cover map for any given time point [19].

In recent years, there has been an increase in the availability of high performance cloud computing. For example, the NASA Earth Exchange (NEX) platform allows the processing and analysis of NASA earth observation data [20]. Amazon Web Service (AWS) also now provides access to the Landsat data archive, enabling analysis of this dataset on the cloud. Google Earth Engine (GEE) is a new high-performance computing platform which gives access to a vast and growing amount of earth observation data as well as processing power to analyze these data at planetary scale.

The launch of these high-performance cloud computing platforms has opened the door to global-scale geospatial data storage, processing and analyzing at a low cost and efficient manner in the cloud [7, 19, 21]. One of the first global scale applications of the Landsat data archive was a study by Hansen et al, which used Landsat satellite data and machine learning to map global forest cover over the 2000–2012 period [7]. Although the focus of their study was to quantify global scale changes in forest cover, theirs is the only recent effort to apply high spatial resolution (30 m) Landsat satellite observation data for mapping global scale land cover over a long period of time (i.e. from 2000–2012). In our study, we present the use of Landsat satellite observation data and GEE cloud platform to map land cover and impervious surface changes over continental Africa for 2000–2015.

## Methods

### 2.1 Earth observation data

In this study, Landsat 7 Enhanced Thematic Mapper Plus (ETM +) surface reflectance data which was computed using the Landsat Ecosystem Disturbance Adaptive Processing System (LEDAPS) were used [22]. A cloud screening algorithm was applied using quality assessment (QA) bands in order to remove snow and cloud contaminated pixels for each Landsat image between 1999 and 2016. Annual composites were then produced by taking the median value from images from the target year, plus or minus one year. For example, for the year 2000, pixel values were obtained by calculating the median of all cloud-free pixels from images between January 1^st^, 1999 and December 31^st^, 2001. A three-year window was used to ensure that at least one cloud-free pixel was available for each annual composite. Similarly, in addition to producing annual raw image composites, annual normalized difference vegetation index (NDVI) and normalized difference water index (NDWI) were computed by taking the median value from 3-year windows. Additionally, annual inter-calibrated night-time light images were used from the National Oceanic and Atmospheric Administration (NOAA) [23–25].

### 2.2. Training data

**Fig 1:**
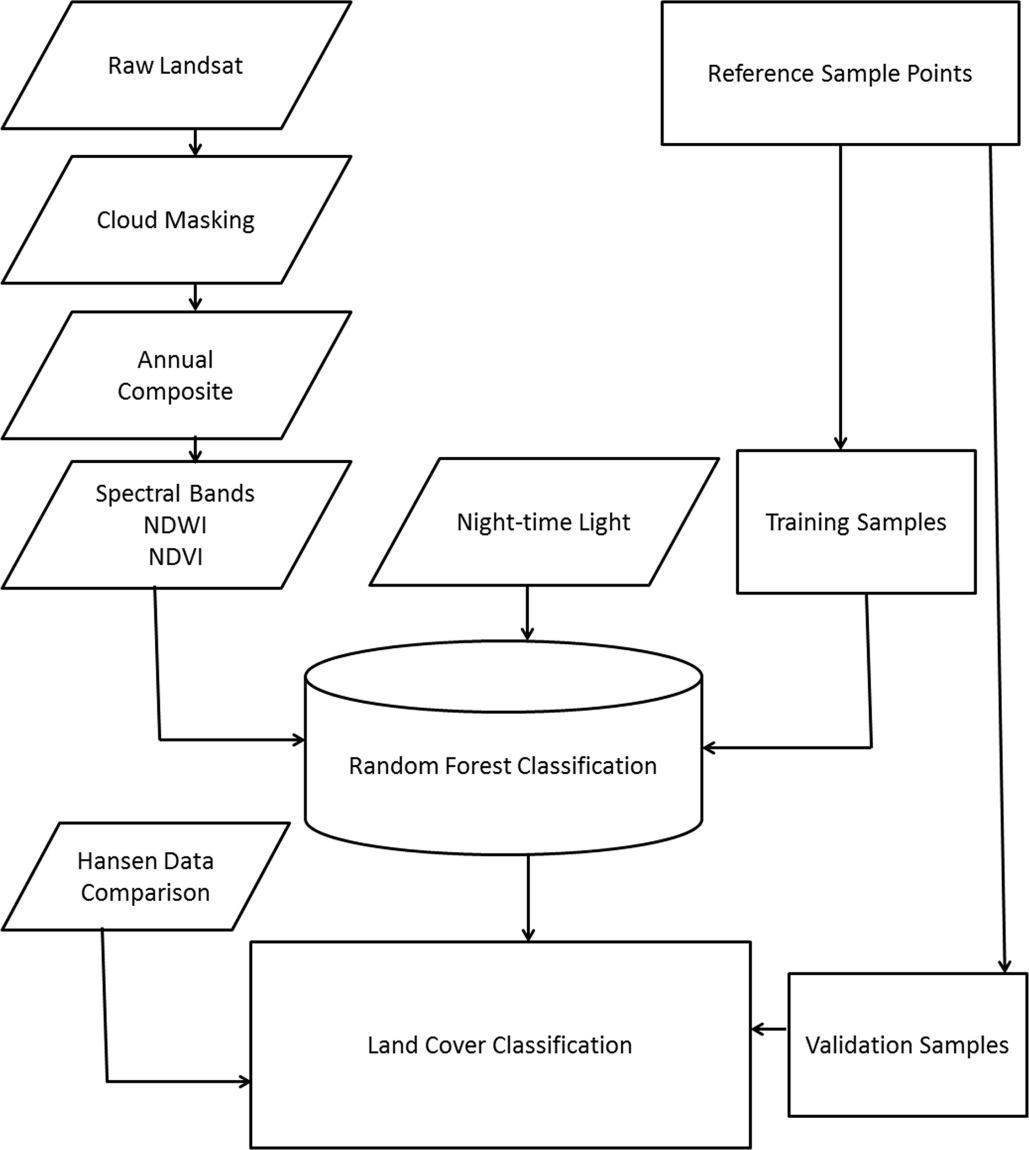
A conceptual model for the land use land cover classification model.

As used by a number of other studies [8, 26–28], training data were derived from visual inspection of freely available high spatial resolution imagery. Recently captured (2015–2016) high-resolution satellite imagery were visually inspected and used to identify Landsat pixels that were entirely made up of one of 7 classes (water, impervious surface, high biomass, low biomass, rock, sand and bare soil) to act as training data. Impervious surface class included asphalt roads, concrete, metal roofs and other built infrastructure while low biomass class included crop field, grass, and shrubland. High biomass class consisted of dense forest. In an effort to ensure that these training data were representative of the classes across the continent, we ensured that training data were captured from all 49 countries in continental Africa, with the aim of capturing 1000 training points per class. For model validation purposes, 80% of sample points (5,664) from each class were randomly selected to act as training data, with 20% of sample points (1,420) withheld as a validation dataset (Fig 1).

### 2.3 Modeling

For each of the training and validation data points, Landsat spectral bands, NDVI, NDWI and night-time light from the 2015 layers were extracted to be used as covariates in the model (Fig 1). To model and predict the 7 LULC classes, a random forest decision tree classification algorithm was deployed on GEE. Decision tree classification algorithms have been used widely to classify satellite data for forest cover mapping [28, 29], wetland mapping [8, 26, 30, 31], cropland mapping [32], and land cover mapping [33–36]. A random forest is a nonparametric machine learning method comprised of ensembles of decision trees [37]. The random forest algorithm creates multiple decision trees which classify a random subset of the training data according to the covariate predictors. In our study, we used 500 trees in the random forest classification. The final classification was based on the majority vote from all the trees. To generate final LULC layers across Africa 2000–2015, training and validation data were combined into a single dataset before the model was run, with annual predictions made from this model using the annual covariate layers. Having made annual predictions, the changes in the area represented by each class from 2000 to 2015 across continental Africa were calculated. This also allowed for an exploration of the direction of change for each LULC class from 2000 to 2015.

As well as modeling 7 classes, estimating the likelihood of a given pixel being impervious was a focus of this study. To do this, the 6 non-impervious surface classes in the training and validation data were collapsed into a single class, resulting in a binary outcome of impervious and non-impervious. A random forest model was then applied to the binary outcome. This model was used to predict the probability (i.e. the proportion of times the model voted) that a pixel was impervious. As for predictions of the 7 LULC classes, final predictions across Africa 2000–2015 were made by combining training and validation data before running the model. All analyses were performed on GEE cloud platform.

### 2.4 Model validation

Using the validation data, a confusion matrix was generated to evaluate predictive accuracy across classes as well as overall accuracy. This allowed an assessment of user’s accuracy (the number of correctly classified pixels divided by the total number of pixels predicted within that class) and producer’s accuracy (the number of correctly classified pixels divided by the total number of pixels truly in that class) for each class. For the model focused on impervious surface, an assessment of predictive accuracy was made by calculating both a confusion matrix as well as area under the curve (AUC) statistic. The AUC statistic represents the probability that a randomly selected truly impervious pixel will be ranked higher by the model than a truly non-impervious pixel. As such, it provides a measurement of the discriminatory power of the model.

In addition to validating the model using out of sample predictions, we compared our predictions to maps of percent tree cover generated by Hansen [7]. This dataset was chosen as it was conducted at a comparable spatial (30 m) and temporal (annual) resolution. As the Hansen study was focused on percent tree cover, rather than LULC, this comparison focused on forest cover only. To compare results, we first classified the Hansen product for 2000 in to high biomass (greater than 30% tree cover) and non-high biomass (less than 30% tree cover) using a threshold of 30% tree cover. This threshold was chosen because it is the closest to our high biomass class. We then compared our high biomass class predictions for 2000 to the high biomass class derived from the Hansen data. Furthermore, we compared our 2010 prediction maps with the 2010 Global Land Cover product (GlobeLand30) at 30 meter spatial resolution which was generated using Landsat data [38]. The GlobeLand30 product consists of 10 classes including water bodies, wetland, built-up, cropland, snow, forest, shrubland, grassland, bareland, and tundra. We chose to compare our 2010 land cover product with the 2010 GlobeLand30 product since theirs is available only for years 2000 and 2010.

## Results

### 3.1 Land use land cover classification

A total of 7,084 data points were captured across the 7 classes, resulting in 5,664 training data points and 1,420 validation data points. Table 1 shows the confusion matrix of observed versus predicted values using the withheld validation data. The model achieved an overall accuracy of 88 percent (Table 1). Class specific user’s and producer’s accuracies ranged from 84–94% and 79–96% respectively. Water appeared to be most accurately predicted, with user’s and producer’s accuracy of 94 and 96% respectively, while low biomass was least accurately predicted with user’s and producer’s accuracies of 84 and 79% respectively.

**Table 1:** Accuracy assessment based on comparison of model predictions (left) against observed validation data (top) for 2015.

	Impervious Surface	Low Biomass	High Biomass	Bare Soil	Sand	Rock	Water	Total	User’s Accuracy (%)
Impervious Surface	186	1	0	19	3	1	0	210	89
Low Biomass	2	198	14	11	2	7	3	237	84
High Biomass	0	31	209	0	0	1	0	241	87
Bare Soil	10	12	2	172	2	3	1	202	85
Sand	3	2	0	2	149	10	2	168	89
Rock	2	4	1	0	4	131	2	144	91
Water	0	2	1	2	6	2	205	218	94
Total	203	250	227	206	166	155	213	1420	
Producer’s Accuracy (%)	92	79	92	83	90	85	96		

**Fig 2:**
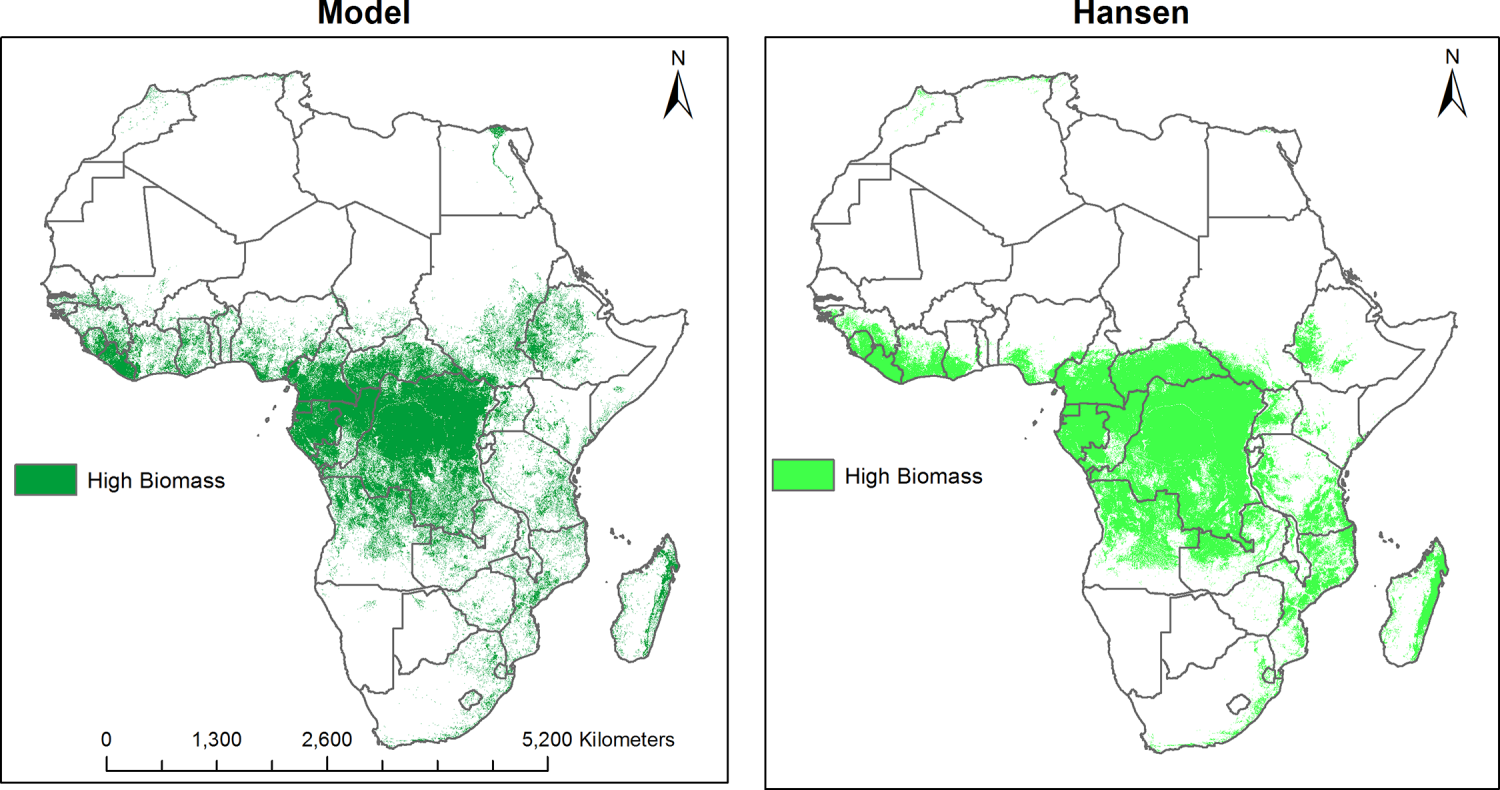
Comparison of predicted high biomass class and Hansen forest cover for 2000.

**Fig 3:**
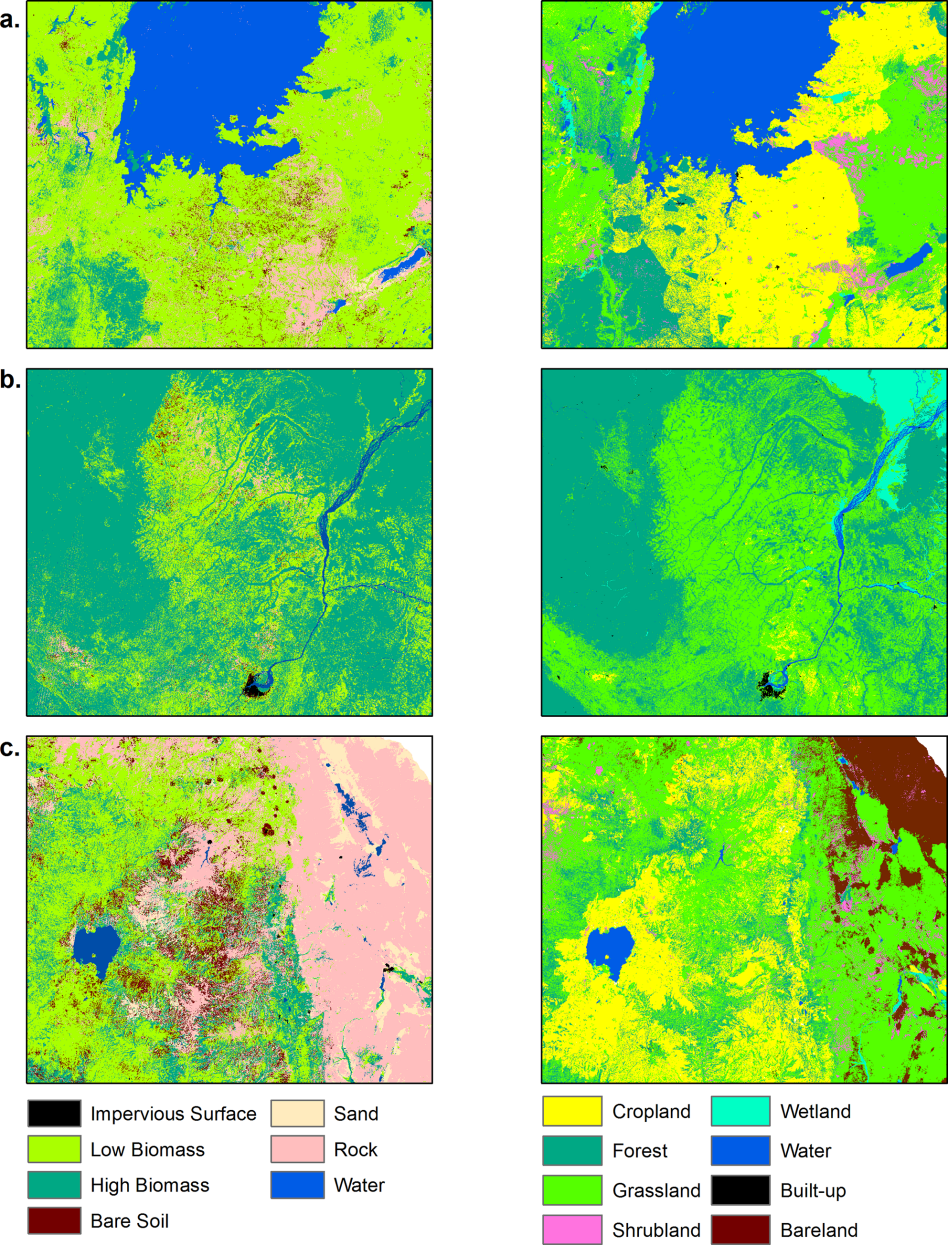
Predicted land cover maps from this study (left) and GlobeLand30 products (right) for the year 2010. (A) Lake Victoria; (B) Congo Basin; (C) Nile Basin.

**Fig 4:**
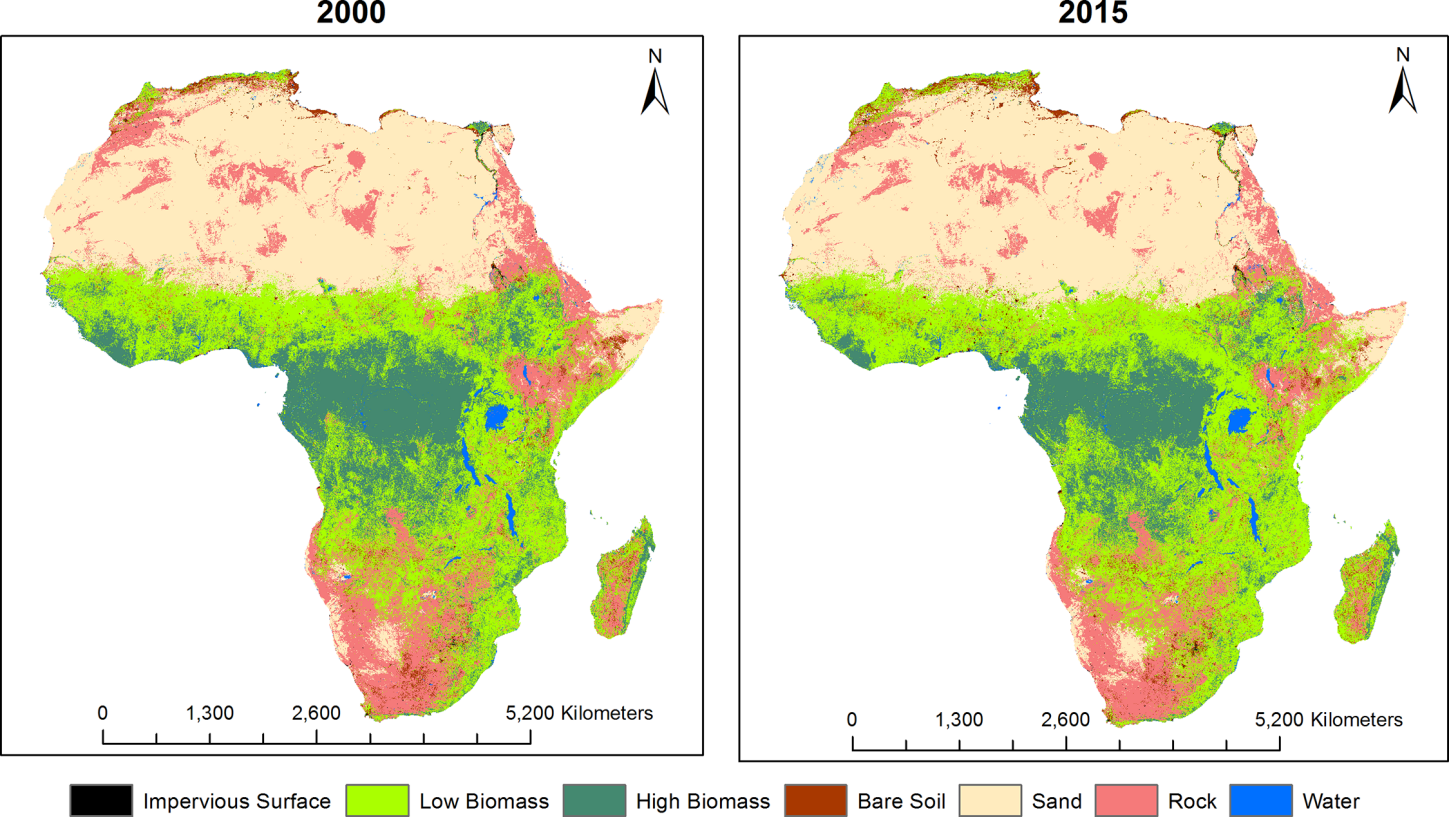
Model predicted land use land cover maps for 2000 and 2015 over Africa.

**Fig 5:**
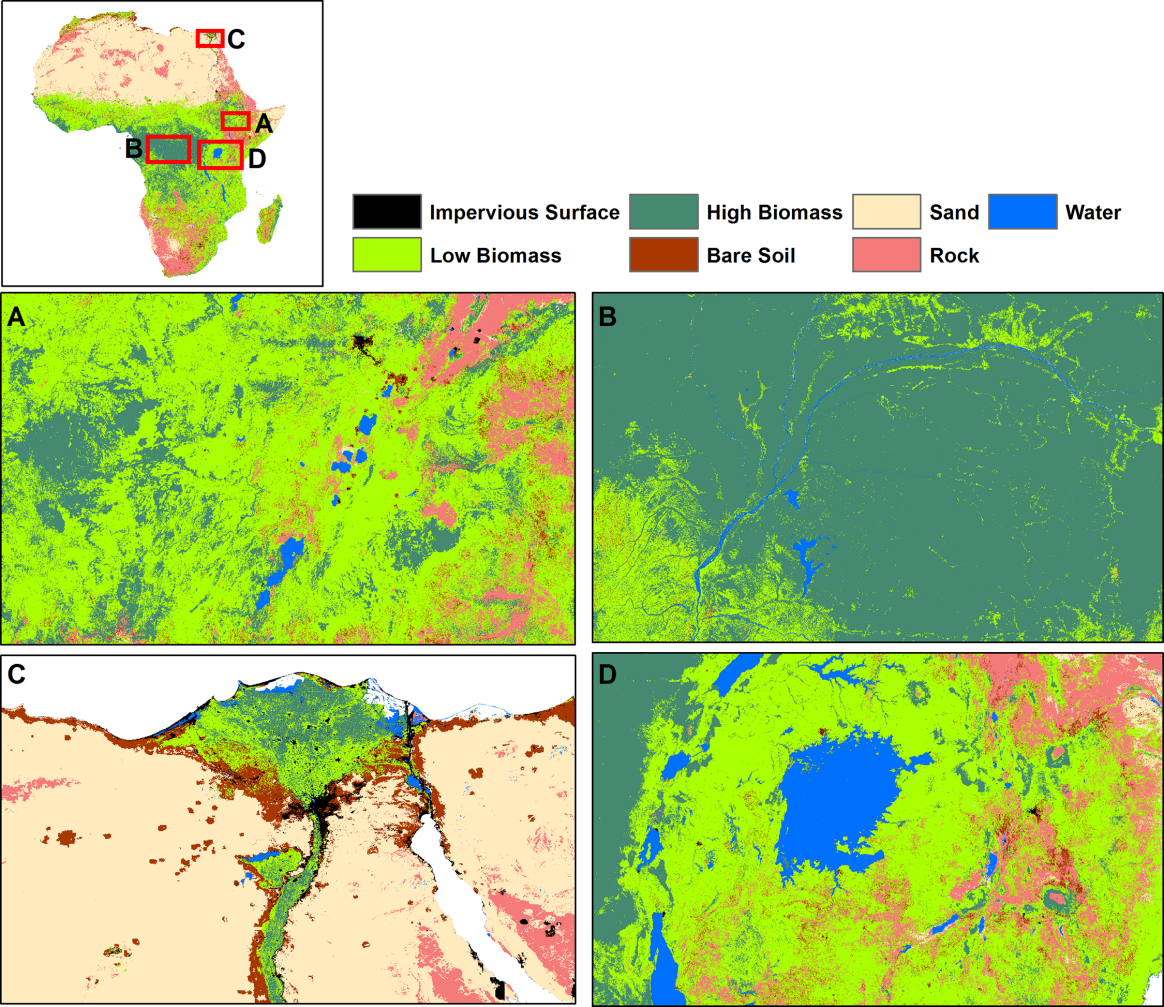
Regional subsets of 2015 land use land cover maps. (A). Rift Valley Lakes; (B) Congo Basin; (C) Nile Delta; (D) Lake Victoria.

Annual LULC predicted maps that consist of seven classes (impervious surface, low biomass, high biomass, bare soil, sand, rock, and water) for the 2000–2015 period were generated based on the random forest classification model. Comparison of high biomass class with the Hansen forest map for the 2000 period showed good agreement. Almost 80% of the total high biomass predicted by our model (4,174,958 Km^2^ out of 5,156,559 Km^2^) matched with the Hansen high biomass class (percent of tree cover greater than 30%) as shown in Fig 2. Generally, there was a good agreement between products in the Congo Basin and western Africa whereas there were mismatches in the East African highlands and Nile Delta regions, with our model predicting more high biomass in these regions. For the 3 sites selected for comparison purposes (Fig 3), a total of 1,465 Km^2^ were classified by the GlobeLand30 as built-up while our model predicted a total 926 Km^2^ as impervious surface for the 2010 period. Additionally, a total of 351,036 Km^2^ were classified as forest by the GlobeLand30 whereas 281,248 Km^2^ were classified as high biomass for the 2010 period by our model. Fig 3 shows visual comparison of our land cover prediction maps with the GlobeLand30 product over three sites including the Lake Victoria, Congo Basin and Nile Basin regions. Fig 4 shows the prediction of the LULC product for the years 2000 and 2015. As shown in Fig 4, there was a reduction in high biomass over the 15 years period throughout continental Africa, with high biomass areas becoming more concentrated around the equatorial belt. Additionally, Fig 5 shows regional subsets of 2015 land use land cover map including the Rift Valley Lakes, Congo Basin, Nile Delta and Lake Victoria.

**Fig 6:**
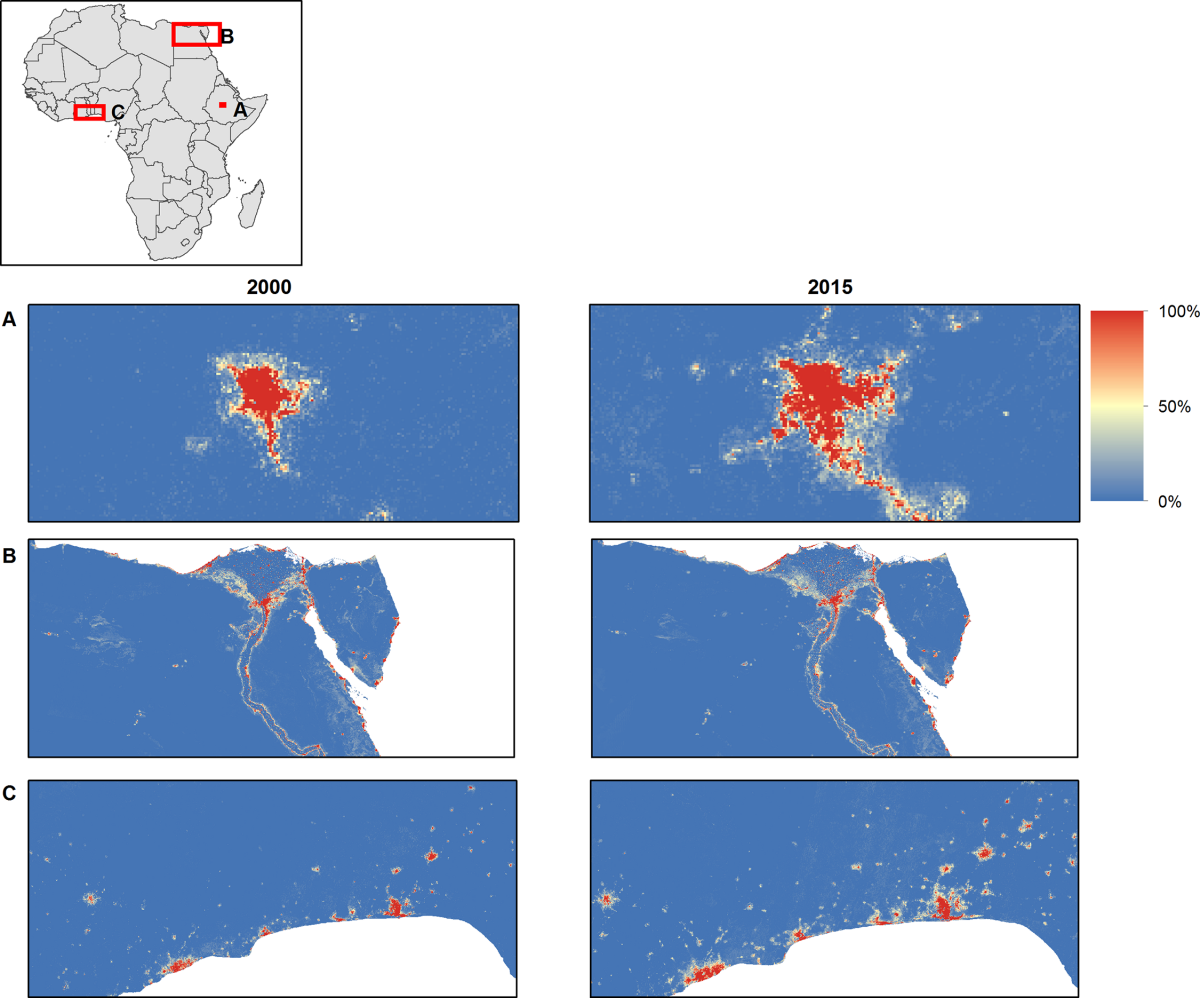
Regional subsets of changes in the probability of impervious surface between 2000–2015. (A). Addis Ababa, Ethiopia; (B) Cairo, Egypt; (C) Lagos, Nigeria.

With regards to the second model focused on impervious surface, an assessment using the validation data indicated that the model showed very high predictive capacity, with an AUC value of 0.98. Fig 6 shows regional subsets of 2015 probability of being impervious including the Rift Valley Lakes, Congo Basin, Nile Delta and Lake Victoria areas.

**Fig 7:**
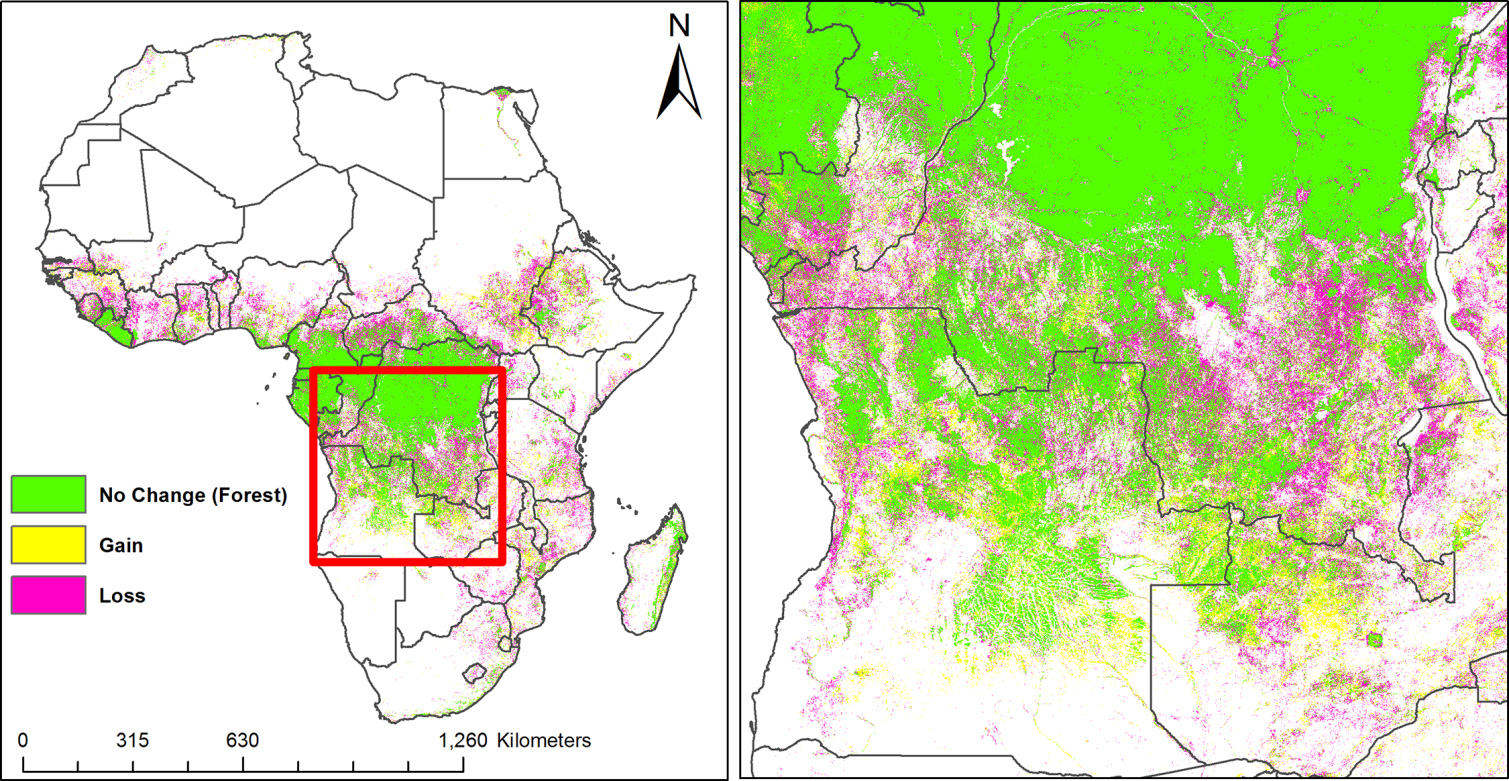
Forest cover change between 2000 and 2015.

Change analysis showed that impervious surface class had the highest relative increase from 2000 to 2015 (38.54%) while high biomass and rock had the highest decreases from 2000 to 2015 with a decrease of around 17% (Fig 7 and Table 2).There was relatively little change in the areas covered by water or sand. An exploration of annual change in the probability of being impervious suggests that changes over the study period were in both directions as shown in four selected regions between 2000 and 2015 (Fig 6). High biomass and rock showed a declining trend whereas impervious surfaces, low biomass, and bare soil showed increasing trend.

**Table 2:** Land use land cover change between 2000 and 2015.

**LULC Class**	2000 (Km^**2**^)	2015 (Km^**2**^)	Total Change (Km^**2**^)	**Total Change (%)**
Impervious Surface	39,436	54,634	15,199	38.5
Low Biomass	9,060,578	10,181,331	1,120,752	12.4
High Biomass	5,156,559	4,261,541	-895,018	-17.4
Bare Soil	681,620	797,121	115,501	16.9
Sand	11,037,403	11,603,370	565,967	5.1
Rock	5,417,149	4,496,410	-920,739	-17.0
Water	306,802	305,140	-1,662	-0.5

**Fig 8:**
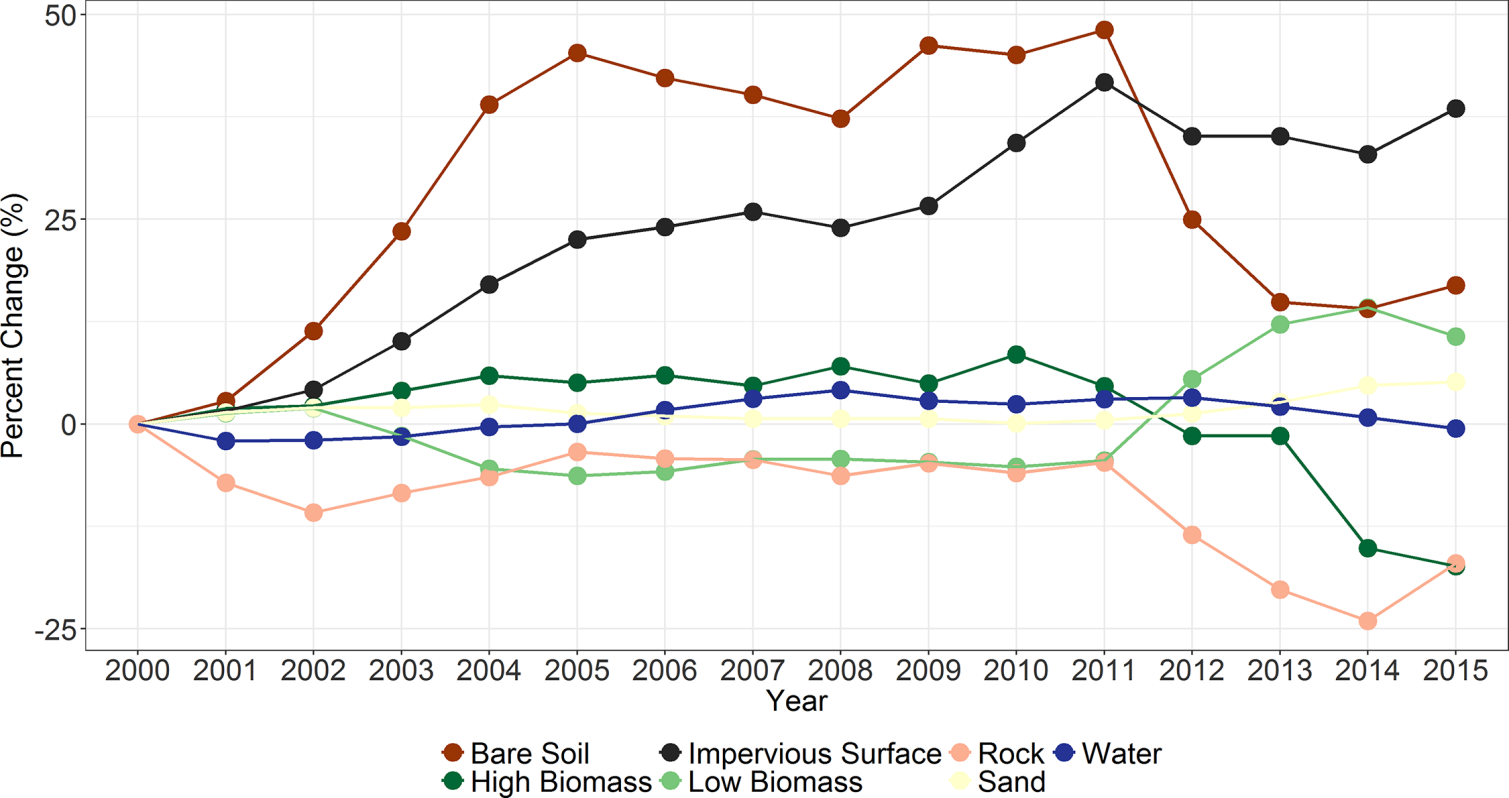
Percentage change in land use land cover classes over Africa 2000–2015.

**Fig 9:**
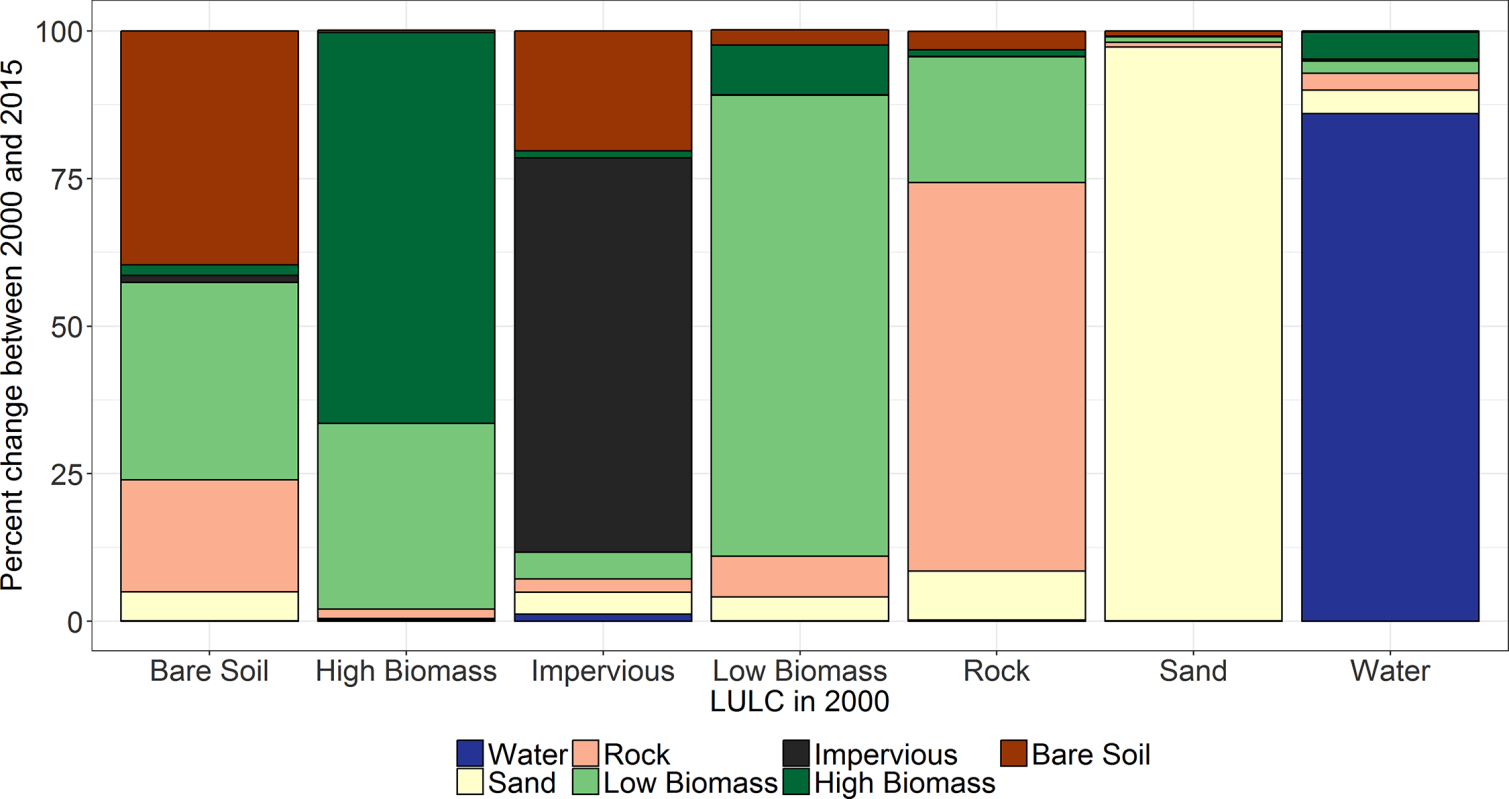
Change of each land use land cover classes from 2000 to 2015.

Growth in the impervious surface was mainly due to conversion from low biomass class to impervious surface. As shown in Figs 8 and 9, sand and water classes were the most stable and did not show substantial change. This study also showed that the decrease in high biomass class was predominantly due to high biomass becoming low biomass (Fig 9).

## Discussion

Regularly updated, high resolution, and continental-scale land cover data are essential for quantifying and monitoring the Earth’s dynamic land surface processes. Despite the free availability of high spatial resolution Landsat satellite data, generation of continental to global scale LULC maps have been challenging due to the need for high-performance computing to store, process and analyze such a large volume of satellite data. As such, earlier efforts to produce land cover products at this scale have been limited to coarse spatial resolution. Platforms such as GEE have opened the door to planetary scale analyses and offer the opportunity to provide a mechanism to continually monitor the Earth’s surface at high spatial and temporal resolution. The utility of GEE to quantify various land surface changes has been demonstrated for forest mapping [7], population mapping [39], cropland mapping [40], and surface water mapping [21]. Here we presented an approach to quantify continental land cover change over a long period of time (15 years) using GEE and Landsat satellite observations.

In the present study, we produced annual LULC maps for continental Africa between 2000 and 2015 showing that the continent has dramatically changed during the study period. Our results indicated area covered by high biomass class showed a declining trend during the study period. These findings were in concordance with Hansen et al who showed a dramatic forest loss between 2000 and 2012 period in subtropical Africa [7]. In an effort to compare our product with existing data, we made comparisons with the Hansen data. While not the same products, a comparative assessment of our high biomass class and the high biomass class generated from the Hansen product (greater than 30% tree cover) for the 2000 period showed 80% agreement. Overall, our product showed very good agreement with the Hansen data. Additionally, comparison of our product with the GlobeLand30 showed good agreement. However, some of our land cover classes and classes from the GlobeLand30 product were different. For example, our low biomass class was represented in three separate classes including wetland, cropland, and grassland in the Globeland30 data. As a result, we focused our comparison with our high biomass class and their forest class as well as our impervious surface and their built-up class. Furthermore, there also appeared to be a large relative increase in man-made impervious surfaces during the 2000–2015 periods. These changes in impervious surface were mostly the conversion of low biomass areas to impervious surfaces. This increase has occurred steadily throughout the period of study, which appears in line with increasing urban growth in Africa [41].

Multi-temporal satellite observations and cloud-based analytic platforms such as GEE could enable cost-effective production of LULC products at low cost and efficient manner. The main advantages of using GEE include reduced processing times and enhanced capacity to perform global scale analysis on high resolution data. There are, however, challenges to using GEE. Some of these challenges include mastery of JavaScript and Python programming languages, limited number of GEE built-in functions, and lack of integration for the current GEE platform with other open source geospatial analytic tools such as R, and QGIS. Although GEE archives a large library of earth observation and geospatial data, data is not available in real-time which may limit its utility to some operational applications that need real-time data access.

While this study suggests that the approach taken here can be used to produce continental LULC products, it should be pointed out that this study has a number of limitations. Firstly, training and validation data were only available for 2015. Having more data points throughout the time period would likely improve the accuracy of the annual maps. This would also allow a better assessment of predictive performance through time. That said, as the spectral signature of these 7 classes is unlikely to have changed through the time period, predictions are likely to be robust. Secondly, the study relied on visual inspection of high-resolution imagery to produce training data; ground truth data from the field were not available. This may have resulted in some misclassification. Additionally, relying on a single image to represent a 1 year period ignores the fact that some training data points may move between classes seasonally (i.e. bare soil in the dry season and low biomass in the wet season). Thirdly, we restricted our analysis to 7 LULC classes which may limit the utility of the current LULC product to some applications. With accurate training data on a wider variety of LULC classes, it may be possible to use the approach described here to produce maps of more than 7 classes used here. Although this study encountered the aforementioned limitations, the standardized approaches demonstrated here and model validation results indicated that the LULC maps presented in this research had high prediction accuracy.

The approach used here to overcome the computational challenges of handling big earth observation data can help scientists and practitioners who lack high-performance computational resources. Future studies can expand on our study and apply our approach to generate global-scale land cover products. The LULC product presented in this study will be freely available (https://geodata.globalhealthapp.net/lulc/) for public use and can be used in other applications to monitor changes in disease transmission, natural resources, biodiversity, and urbanization.

## References

[1] Turner BL, Lambin EF, Reenberg A. The emergence of land change science for global environmental change and sustainability. P Natl Acad Sci USA. 2007;104(52):20666–71. doi: 10.1073/pnas.0704119104 18093934 10.1073/pnas.0704119104 PMC2409212

[2] Pielke RA, Pitman A, Niyogi D, Mahmood R, McAlpine C, Hossain F, et al Land use/land cover changes and climate: modeling analysis and observational evidence. Wires Clim Change. 2011;2(6):828–50. doi: 10.1002/wcc.144

[3] Ramankutty N, Foley JA. Estimating historical changes in global land cover: Croplands from 1700 to 1992. Global Biogeochem Cy. 1999;13(4):997–1027. doi: 10.1029/1999gb900046

[4] Costa MH, Botta A, Cardille JA. Effects of large-scale changes in land cover on the discharge of the Tocantins River, Southeastern Amazonia. J Hydrol. 2003;283(1–4):206–17. doi: 10.1016/S0022-1694(03)00267-1

[5] Hansen MC, Egorov A, Potapov PV, Stehman SV, Tyukavina A, Turubanova SA, et al Monitoring conterminous United States (CONUS) land cover change with Web-Enabled Landsat Data (WELD). Remote Sens Environ. 2014;140:466–84. doi: 10.1016/j.rse.2013.08.014

[6] Hansen MC, Egorov A, Roy DP, Potapov P, Ju JC, Turubanova S, et al Continuous fields of land cover for the conterminous United States using Landsat data: first results from the Web-Enabled Landsat Data (WELD) project. Remote Sens Lett. 2011;2(4):279–88. doi: 10.1080/01431161.2010.519002

[7] Hansen MC, Potapov PV, Moore R, Hancher M, Turubanova SA, Tyukavina A, et al High-Resolution Global Maps of 21st-Century Forest Cover Change. Science. 2013;342(6160):850–3. doi: 10.1126/science.1244693 24233722 10.1126/science.1244693

[8] Midekisa A, Senay GB, Wimberly MC. Multisensor earth observations to characterize wetlands and malaria epidemiology in Ethiopia. Water Resour Res. 2014;50(11):8791–806. doi: 10.1002/2014WR015634 25653462 10.1002/2014WR015634 PMC4303930

[9] Patz JA, Norris DE. Land use change and human health. Geoph Monog Series. 2004;153:159–67. doi: 10.1029/153gm13

[10] Arino O, Bicheron P, Achard F, Latham J, Witt R, Weber JL. GLOBCOVER The most detailed portrait of Earth. Esa Bull-Eur Space. 2008;(136):24–31.

[11] Friedl MA, Sulla-Menashe D, Tan B, Schneider A, Ramankutty N, Sibley A, et al MODIS Collection 5 global land cover: Algorithm refinements and characterization of new datasets. Remote Sens Environ. 2010;114(1):168–82. doi: 10.1016/j.rse.2009.08.016

[12] Hansen MC, Defries RS, Townshend JRG, Sohlberg R. Global land cover classification at 1km spatial resolution using a classification tree approach. Int J Remote Sens. 2000;21(6–7):1331–64. doi: 10.1080/014311600210209

[13] Loveland TR, Reed BC, Brown JF, Ohlen DO, Zhu Z, Yang L, et al Development of a global land cover characteristics database and IGBP DISCover from 1 km AVHRR data. Int J Remote Sens. 2000;21(6–7):1303–30. doi: 10.1080/014311600210191

[14] Wulder MA, Masek JG, Cohen WB, Loveland TR, Woodcock CE. Opening the archive: How free data has enabled the science and monitoring promise of Landsat. Remote Sens Environ. 2012;122:2–10. doi: 10.1016/j.rse.2012.01.010

[15] DeVries B, Verbesselt J, Kooistra L, Herold M. Robust monitoring of small-scale forest disturbances in a tropical montane forest using Landsat time series. Remote Sens Environ. 2015;161:107–21. doi: 10.1016/j.rse.2015.02.012

[16] Dong JW, Xiao XM, Kou WL, Qin YW, Zhang GL, Li L, et al Tracking the dynamics of paddy rice planting area in 1986–2010 through time series Landsat images and phenology-based algorithms. Remote Sens Environ. 2015;160:99–113. doi: 10.1016/j.rse.2015.01.004

[17] Schroeder TA, Healey SP, Moisen GG, Frescino TS, Cohen WB, Huang CQ, et al Improving estimates of forest disturbance by combining observations from Landsat time series with US Forest Service Forest Inventory and Analysis data. Remote Sens Environ. 2014;154:61–73. doi: 10.1016/j.rse.2014.08.005

[18] Zhu Z, Woodcock CE, Holden C, Yang ZQ. Generating synthetic Landsat images based on all available Landsat data: Predicting Landsat surface reflectance at any given time. Remote Sens Environ. 2015;162:67–83. doi: 10.1016/j.rse.2015.02.009

[19] Giri C, Pengra B, Long J, Loveland TR. Next generation of global land cover characterization, mapping, and monitoring. Int J Appl Earth Obs. 2013;25:30–7. doi: 10.1016/j.jag.2013.03.005

[20] Nemani R. Nasa Earth Exchange: Next Generation Earth Science Collaborative. Int Arch Photogramm. 2011;38-8(W20):17–.

[21] Donchyts G, Baart F, Winsemius H, Gorelick N, Kwadijk J, van de Giesen N. Earth's surface water change over the past 30 years. Nat Clim Change. 2016;6(9):810–3.

[22] Masek JG, Vermote EF, Saleous NE, Wolfe R, Hall FG, Huemmrich KF, et al A Landsat surface reflectance dataset for North America, 1990–2000. Ieee Geosci Remote S. 2006;3(1):68–72. doi: 10.1109/Lgrs.2005.857030

[23] Mellander C, Lobo J, Stolarick K, Matheson Z. Night-Time Light Data: A Good Proxy Measure for Economic Activity? Plos One. 2015;10(10). doi: 10.1371/journal.pone.0139779 26496428 10.1371/journal.pone.0139779 PMC4619681

[24] Noor AM, Alegana VA, Gething PW, Tatem AJ, Snow RW. Using remotely sensed night-time light as a proxy for poverty in Africa. Popul Health Metr. 2008;6:5 doi: 10.1186/1478-7954-6-5 ; PubMed Central PMCID: PMC2577623. 18939972 10.1186/1478-7954-6-5 PMC2577623

[25] Savory DJ, Andrade-Pacheco R, Gething PW, Midekisa A, Bennett A, Sturrock HJW. Intercalibration and Gaussian Process Modeling of Nighttime Lights Imagery for Measuring Urbanization Trends in Africa 2000–2013. Remote Sens-Basel. 2017;9(7). doi: 10.3390/rs9070713

[26] Bwangoy JRB, Hansen MC, Roy DP, De Grandi G, Justice CO. Wetland mapping in the Congo Basin using optical and radar remotely sensed data and derived topographical indices. Remote Sens Environ. 2010;114(1):73–86. doi: 10.1016/j.rse.2009.08.004

[27] Dong JW, Xiao XM, Sheldon S, Biradar C, Duong ND, Hazarika M. A comparison of forest cover maps in Mainland Southeast Asia from multiple sources: PALSAR, MERIS, MODIS and FRA. Remote Sens Environ. 2012;127:60–73. doi: 10.1016/j.rse.2012.08.022

[28] Hansen MC, Roy DP, Lindquist E, Adusei B, Justice CO, Altstatt A. A method for integrating MODIS and Landsat data for systematic monitoring of forest cover and change in the Congo Basin. Remote Sens Environ. 2008;112(5):2495–513. doi: 10.1016/j.rse.2007.11.012

[29] DeFries R, Hansen M, Steininger M, Dubayah R, Sohlberg R, Townshend J. Subpixel forest cover in central Africa from multisensor, multitemporal data. Remote Sens Environ. 1997;60(3):228–46. doi: 10.1016/S0034-4257(96)00119-8

[30] Tulbure MG, Broich M. Spatiotemporal dynamic of surface water bodies using Landsat time-series data from 1999 to 2011. Isprs J Photogramm. 2013;79:44–52. doi: 10.1016/j.isprsjprs.2013.01.010

[31] Wright C, Gallant A. Improved wetland remote sensing in Yellowstone National Park using classification trees to combine TM imagery and ancillary environmental data. Remote Sens Environ. 2007;107(4):582–605. doi: 10.1016/j.rse.2006.10.019

[32] Yan L, Roy DP. Improved time series land cover classification by missing-observation-adaptive nonlinear dimensionality reduction. Remote Sens Environ. 2015;158:478–91. doi: 10.1016/j.rse.2014.11.024

[33] Clark ML, Kilham NE. Mapping of land cover in northern California with simulated hyperspectral satellite imagery. Isprs J Photogramm. 2016;119:228–45. doi: 10.1016/j.isprsjprs.2016.06.007

[34] Gounaridis D, Koukoulas S. Urban land cover thematic disaggregation, employing datasets from multiple sources and RandomForests modeling. Int J Appl Earth Obs. 2016;51:1–10. doi: 10.1016/j.jag.2016.04.002

[35] Rodriguez-Galiano VF, Ghimire B, Rogan J, Chica-Olmo M, Rigol-Sanchez JP. An assessment of the effectiveness of a random forest classifier for land-cover classification. Isprs J Photogramm. 2012;67:93–104. doi: 10.1016/j.isprsjprs.2011.11.002

[36] Gessner U, Machwitz M, Esch T, Tillack A, Naeimi V, Kuenzer C, et al Multi-sensor mapping of West African land cover using MODIS, ASAR and TanDEM-X/TerraSAR-X data. Remote Sens Environ. 2015;164:282–97. doi: 10.1016/j.rse.2015.03.029

[37] Breiman L. Random forests. Mach Learn. 2001;45(1):5–32. doi: 10.1023/A:1010933404324

[38] Chen J, Chen J, Liao AP, Cao X, Chen LJ, Chen XH, et al Global land cover mapping at 30 m resolution: A POK-based operational approach. Isprs J Photogramm. 2015;103:7–27. doi: 10.1016/j.isprsjprs.2014.09.002

[39] Patel NN, Angiuli E, Gamba P, Gaughan A, Lisini G, Stevens FR, et al Multitemporal settlement and population mapping from Landsat using Google Earth Engine. Int J Appl Earth Obs. 2015;35:199–208. doi: 10.1016/j.jag.2014.09.005

[40] Xiong J, Thenkabail PS, Gumma MK, Teluguntla P, Poehnelt J, Congalton RG, et al Automated cropland mapping of continental Africa using Google Earth Engine cloud computing. Isprs J Photogramm. 2017;126:225–44. doi: 10.1016/j.isprsjprs.2017.01.019

[41] Linard C, Tatem AJ, Gilbert M. Modelling spatial patterns of urban growth in Africa. Appl Geogr. 2013;44:23–32. doi: 10.1016/j.apgeog.2013.07.009 25152552 10.1016/j.apgeog.2013.07.009 PMC4139116

